# The Proofs That Lithotrity Is an Eminently Successful Operation

**Published:** 1865-07

**Authors:** Henry Thompson

**Affiliations:** Surgeon Extraordinary to H.M. the King of the Belgians, Surgeon to University College Hospital


### THE PROOFS THAT LITHOTRITY IS AN EMINENTLY SUCCESSFUL OPERATION.

By HENRY THOMPSON, F.R.C.S., Surgeon Extraordinary to H.M. the King of the Belgians, Surgeon to University College Hospital.

I am induced to offer a few remarks on this subject, partly because a statement has recently appeared in *the Lancet*, made by my friend Mr. Holmes Coote, to the effect that "the general impression formed respecting the relative merit of the two operations"—namely lithotomy and lithotrity—is highly unfavorable to the latter, for "the dangers attending lithotrity are greater than are commonly supposed; and that the very favorable returns quoted by some surgeons proceed from their considering each 'sitting' as a completed case, whereas the result should be regarded only after the patient has been freed of his complaint."

One portion of this extract must be noticed first and briefly. If any surgeons are in the habit of representing each of the several sittings required to remove a stone as a completed case, there is but one term that can characterize such conduct, and it is a term that I dare to employ, only because I do not believe there are any to whom it is in the least degree applicable. Such conduct would simply be grossly fraudulent. Any person adopting it would state that he had operated on five, seven, or ten patients (or whatever number of sittings might have been necessary to free any given patient from his stone), when in truth he had operated only on one. I have had to examine many statements in relation to this subject, but I have never had reason to suspect such a practice. Several weeks have elapsed since the suggestion was made, and, observing that at present it has elicited no remark, I felt that it could not continue longer unnoticed.

But, secondly, in relation to the important question of the dangers or the success of lithotrity, I confess that it is necessary to differ very widely from the opinion which has been said to be general. I think I shall be able to show that lithotrity is a safe and efficient operation for other cases than those belonging to the very narrow category to which Mr. Coote would confine it—viz., "almost exclusively to cases of men in middle or advanced life, of gouty habit, and with a deep perineum, in whom, after intervals of a few months, a small uric-acid calculus drops from the kidney into the bladder, the kidneys being in their general structure healthy." It is further enjoined that this calculus is to be crushed "while it is yet small." Further still—"the lithotrite should, under no circumstances, be used for the purpose of crushing an oxalate-of-lime calculus."

With the object of aiding the practical solution of this question, a group of consecutive cases of lithotrity will be presented with their results. I shall, in short, adduce my own personal experience of the operation during the year just closed; that is, a brief statement of every case in which I have performed and completed lithotrity between Jan. 1st and Dec. 31st, 1864. And, surely, it will be admitted that whether this *resume* affords an exceptionally good result or not (and I contend that it does not), the possibility of such a result suffices to prove the extreme value of the operation for a very considerable range of cases. The patients on whom I have so operated during 1864, are nineteen in number; and I think the result may be regarded, although but a small contribution, yet as numerically much more valuable for inferential purposes than so small a number as four cases, of which two were fatal, which furnished the theme for Mr. Coote's remarks. As will appear, not one of mine was so; in one case only the operation was followed by death in a few weeks, opinions respecting the cause of which may be various; the others made good recoveries.

I ought to premise that, having personally no choice as to the performance of the cutting or of the crushing operation, I have been ready to apply either, according to what, after careful consideration of all the circumstances of the case, appeared to be the best for it. Lastly, I have refused to operate in any way upon one case only—one in which the last chance of affording relief had wholly disappeared. Favorable cases, therefore, have not been selected for either proceeding. That there may be no error, I shall in each case name one individual who has seen it with me, so that a guarantee is afforded of the accuracy of my statement. Without any fear that it would be called in question, I still prefer this as the best rule to adopt, since it places the facts stated beyond the suspicion of error, and within the reach of present inquiry.

CASE 1. A gentleman, aged seventy-four. Uric-acid stone; medium size.* Seven sittings. January and February, 1864;

* By the term "medium," I imply (as elsewhere stated) a stone which measures about an inch in diameter in the lithotrite. The terms "small" and "large," denote stones relatively much smaller and larger than this standard.

seen by Mr. Clover, who gave him chloroform at the patient's special request at first sitting, but was not repeated. Success perfect.

CASE 2. A gentleman, aged sixty-nine. Two uric-acid stones of considerable size. Eighteen sittings. Spring, 1864. A patient of Mr. Gayland, of Devonshire-street, who was present on every occasion. Success perfect.

CASE 3. A gentleman, aged forty-five. Uric-acid and urates, full medium size. Eight sittings. Spring, 1864. Dr. Koepl, of Brussels, who was then in town, was present at two sittings. Success perfect.

CASE 4. A gentleman, aged forty-six. Oxalate of lime; small medium size. Five sittings. Spring, 1864. Dr. Pancoast, of Philadelphia, then in town, was present at one or two of the sittings. Success perfect.

CASE 5. A gentleman, aged seventy-six. A smallish phosphatic stone. Five sittings. April and May, 1864. This case was seen by Dr. Jenner two or three times. Success perfect.

CASE 6. A gentleman, aged forty-eight. A medium-sized uric-acid stone. Six sittings. Mr. Hilton was present at one sitting. Success perfect.

CASE 7. A gentleman, aged seventy. Large mixed calculus. Eleven sittings. Summer, 1864. Seen two or three times by Mr. M. B. Hill. All symptoms of stone disappeared. Some trouble remains with pre-existing enlarged prostate; but he rides, drives, and is actively employed.

CASE 8. A gentleman, aged sixty-eight. A rather small calculus, mixed. Five sittings. Summer, 1864. M. Harris, of Gower-street, was present at every sitting. Success perfect.

CASE 9. A gentleman, aged forty-eight. A rather large uric-acid stone. Eleven sittings. Summer, 1864. Mr. Hilton was present two or three times. Success perfect.

CASE 10. A gentleman, aged sixty-nine. Small uric-acid stone. Five sittings. Dr. Koepl was present at the first. The symptoms disappeared, and he returned home. A fortnight afterwards the patient called on me to complain of a little return of pain. I advised waiting a week or two before sounding. This was at the beginning of June. At the end of the month he had severe rigors and fever, no instruments having been used. He gradually sank in the middle of July. At the autopsy, a calculus, the size of a bean, and smaller ones, were found in the right kidney; in the left was a large abscess. In the bladder there were four calculi, like those in the kidney, which had probably recently descended. The operation was incapable of removing the graver part of the malady—viz., that of the kidneys.

Case 11. A gentleman, aged sixty-nine. Large uric-acid stone. Ten sittings. Summer, 1864. Mr. M. B. Hill saw this patient frequently; my friend Mr. Norman, of Southsea, also, on one occasion. Success perfect.

Case 12. A gentleman, aged sixty-two. Autumn, 1864. Small uric-acid stone. Three sittings. Mr. Hammerton was present each time. Success perfect.

Case 13. A gentleman, aged sixty-two. . Autumn, 1864. Large calculus, uric-acid, and urates. Thirteen sittings. Dr. Harley was present at one sitting. Success perfect.

Case 14. A gentleman, aged forty-nine. Small uric-acid stone. One sitting. Mr. Clippingdale saw this case with me, in Nov., 1864. A subsequent severe illness, from which he slowly recovered; but he writes me now, that he has "no return of the old complaint."

Case 15. A man, aged fifty-nine; University College Hospital. Rather small uric-acid stone. Eight sittings. Nov. and Dec., 1864. The calculus could never be found or seized in any other way than with the blades of the lithotrite directed downwards immediately behind the prostate, however high the pelvis was raised. Discharged free from all symptoms.

Case 16. A boy, aged six; University College Hospital. Small uric-acid stone, the size of a small bean, crushed at one sitting. Discharged cured Dec., 1864.

Case 17. A gentleman, aged seventy-five. A rather small phosphatic stone, broken up and removed at one sitting in Dec., 1864. Mr. Tatham, of Brighton, was present. The patient had long passed no urine, except by catheter. This condition of course remains, minus the symptoms of stone.

Case 18. A gentleman aged fifty-seven. A small oxalate of lime calculus, crushed at one sitting in Nov., 1864. Mr. M. B. Hill was present. Successful as far as the removal of the stone is concerned. The patient is still subject to the formation of small calculi, which he has passed in great number.

Case 19. A gentleman, aged fifty-eight. A uric-acid stone of rather more than medium size. Five sittings. Dec., 1864. Mr. P. Jackson, and Mr. C. Jackson, of Wimpole-street were present at each sitting. Success perfect.

This list comprises eighteen adults, all relieved of stones, of which but a few were small. One patient only died, and certainly not as a direct result of lithotrity, but of renal disease and renal calculus, after having recovered from the primary effects of the operation. Of these eighteen adults, the youngest was forty-five years old, the eldest seventy-five; the majority were above sixty-two years; four were seventy and upwards; and the average age of the whole number is also, as nearly as possible, sixty-two years. No one, I presume, will imagine that for these cases the operation of lithotomy would have afforded results in any way comparable; nevertheless, many of the stones were of considerable size.

I venture to add that, in my opinion, the success of lithotrity depends upon the method adopted. During a considerable period of the history of that operation, its results were undoubtedly inferior to the results of lithotomy in average hands; and if the same method be still employed, no better results can be expected. I propose to add a few remarks on what appears to me to constitute the safe and successful practice of lithotrity in the succeeding number.—*London Lancet.*

